# The Combined Effects of Nicotine and Cannabis on Cortical Thickness Estimates in Adolescents and Emerging Adults

**DOI:** 10.3390/brainsci14030195

**Published:** 2024-02-21

**Authors:** Margie Hernandez Mejia, Kelly E. Courtney, Natasha E. Wade, Alexander Wallace, Rachel E. Baca, Qian Shen, Joseph Patrick Happer, Joanna Jacobus

**Affiliations:** 1 San Diego State University/University of California San Diego Joint Doctoral Program in Clinical Psychology, San Diego, CA 92182, USA; 2 Department of Psychiatry, University of California, San Diego, CA 92093, USA

**Keywords:** adolescence, young adults, nicotine, cannabis, cortical thickness

## Abstract

Early life substance use, including cannabis and nicotine, may result in deleterious effects on the maturation of brain tissue and gray matter cortical development. The current study employed linear regression models to investigate the main and interactive effects of past-year nicotine and cannabis use on gray matter cortical thickness estimates in 11 bilateral independent frontal cortical regions in 223 16–22-year-olds. As the frontal cortex develops throughout late adolescence and young adulthood, this period becomes crucial for studying the impact of substance use on brain structure. The distinct effects of nicotine and cannabis use status on cortical thickness were found bilaterally, as cannabis and nicotine users both had thinner cortices than non-users. Interactions between nicotine and cannabis were also observed, in which cannabis use was associated with thicker cortices for those with a history of nicotine and tobacco product (NTP) use in three left frontal regions. This study sheds light on the intricate relationship between substance use and brain structure, suggesting a potential modulation of cannabis’ impact on cortical thickness by nicotine exposure, and emphasizing the need for further longitudinal research to characterize these interactions and their implications for brain health and development.

## 1. Introduction

In the ever-changing landscape of youth substance use, the co-use of nicotine and cannabis is a major concern. Nicotine tobacco products (NTPs), including cigarettes, smokeless tobacco, and vaping devices, play a significant role in this trend. Data from the 2022 US Monitoring the Future survey report past-year cannabis use at 31% and past-year nicotine vaping at 27% among 12th-grade students [1], while data for 19–22-year-olds shows past-year cannabis use at 41% and nicotine vaping rates of 26–30% [2]. While this study highlights individual prevalence rates for past-year cannabis and nicotine use, it does not provide information on potential overlap between users, underscoring the need for ongoing research in this area. Over the past two decades, cannabis users have been more likely to use tobacco and vice versa [3,4,5,6,7,8,9,10]. A study involving high school students in the U.S. revealed that 59% self-reported using both nicotine and cannabis in the past 30 days [11]. Another U.S.-focused study focused on 15–22-year-olds found that those who had used cigarettes were 3.2 times more likely to report past 30-day use of vaporized cannabis, and 5.5 times more likely to report combustible marijuana use in California [12]. These findings underscore the overlap between cannabis and tobacco use in adolescence and young adulthood.

The period of late adolescence into young adulthood is associated with dynamic brain development, including gray matter refinement, protracted white matter development, and reward system circuit changes [13,14,15,16,17,18,19,20]. As the brain’s reward, motivation, and decision-making areas continue evolving, young individuals become more susceptible to the impact of addictive substances such as nicotine and cannabis, likely driven by hyperactivation of reward systems, greater salience attribution, and habit formation [21,22]. Studies investigating the impact of cannabis use on the developing brain have suggested a range of structural and functional changes. Brain imaging studies, encompassing both cross-sectional and longitudinal designs, have reported alterations in cortical thickness estimates, reduced white matter integrity, and reduced cortical surface area among 16–22-year-olds who use cannabis, in comparison to non-users [23,24,25,26]. Similarly, investigations into the effects of nicotine on the developing brain suggest variations in gray and white matter integrity in comparison to non-users. For example, adolescent and young adult cigarette smokers, as opposed to non-smokers, have exhibited a reduction in frontal cortical thickness, an increase in subcortical striatal volume, thinner gray volume, and greater subcortical surface morphometry [27,28,29,30].

The concurrent use of cannabis and nicotine during the critical phase of adolescent brain development raises concerns regarding the potential additive or synergistic effects of the substances, which could amplify their individual impacts. Research has highlighted distinct neurobehavioral relationships in individuals using both substances. Notably, smaller hippocampal volumes have been linked to better memory performance among co-users, in contrast to a control group, where larger hippocampal volumes were correlated with improved memory scores [31]. Additionally, the interaction between cannabis and nicotine and tobacco product (NTP) use may also be compensatory, where one substance may offset or mitigate the effects of the other [28,32]. Interactive and compensatory relationships have been noted in cognitive functioning, including episodic and working memory [33,34,35], as well as white matter microstructure and cerebral blood flow [36,37], suggesting nicotine’s potential to mitigate cannabis-related impairment. Nonetheless, in a study comparing gray matter volumes, cannabis, tobacco, and co-users showed increased volume in the left putamen but decreased volumes in the left cerebellum and thalamus compared to healthy controls, with tobacco users resembling healthy controls in thalamic volume [38].

While preliminary evidence suggests a potential compensatory relationship between NTPs and cannabis on markers of brain tissue health (e.g., white matter integrity cerebral blood flow) [28,36,37,39], our current understanding of how co-use patterns specifically affect brain structure, neural circuits, and neurobiological development remains limited. Notably, there has been a paucity of prior research investigating the interactive effects of NTPs and cannabis on gray matter tissue integrity in adolescents and emerging adults, with only one study to the best of our knowledge [39]. Gray matter, a crucial component of the brain, undergoes intricate changes during this developmental period, encompassing processes like synaptic pruning, cortical thinning, and new synaptic formations [18,40]. Understanding the interplay between nicotine and cannabis and the development of gray matter is essential for assessing potential consequences on overall brain structure and function, including cognition and emotional regulation. This study aimed to examine the individual and combined effects of NTPs (with n = 136; without n = 87) and cannabis (with n = 156; without n = 67) on 11 bilateral independent frontal gray matter cortical regions among participants aged 16–22.

We hypothesized that: (1) there would be an independent and positive association between nicotine use and cortical thickness measurements, as indicated by previous research [27,29]; (2) there would be an independent and positive association between cannabis use and cortical thickness measurements [15,41,42]; and (3) given our preliminary research showing the potential for a compensatory effect of nicotine on cannabis-related neural outcomes [36,37], we also hypothesized that nicotine use over the past year would moderate the effect of cannabis on neural-related outcomes such that the effects of cannabis on cortical thickness estimates would be less robust among nicotine users than non-users.

## 2. Materials and Methods

### 2.1. Participants and Procedures

This study included N = 223 participants aged 16 to 22, drawn from an investigation focused on exploring the impact of concurrent cannabis and NTP use on brain health. Recruitment of participants occurred through the distribution of flyers, in physical form and/or electronically, across local high schools, community colleges, four-year universities, and social media platforms (i.e., Facebook and Instagram). A 5–10 min phone screening interview was administered to individuals who expressed interest in participating after reviewing recruitment materials. The interview’s objectives were to provide a brief overview of the study, including its purpose, procedures, and confidentiality while verifying whether potential participants met the inclusion and exclusion criteria outlined below. The study was approved by the University of California San Diego Human Research Protections Program, and all participants went through the formal process of written informed consent. For participants under 18 years of age, parental consent and participant assent were obtained.

Inclusion criteria for enrollment were determined by past-month self-reported substance use: (1) exclusive cannabis use (averaging ≥ 1×/week), (2) exclusive use of NTPs (averaging ≥ 1×/week), (3) concurrent use of both cannabis and NTPs (averaging ≥ 1×/week for both cannabis and nicotine), and (4) minimal to no use of either substance (≤15 episodes of cannabis and of NTPs in the past 6 months). Enrollment groups were defined to ensure variability in recency of substance use for those enrolled in the study but were not the primary focus of this investigation. Participants who were excluded from the study had one or more of the following characteristics: current or past DSM-5 psychiatric disorder, excluding cannabis and/or tobacco use disorder; use of any illicit substance, apart from alcohol, NTPs, or cannabis, exceeding 10 times; presence of acute influence of alcohol or cannabis use during the study visit, confirmed via toxicology assessments (e.g., breathalyzer, urine, or oral fluid tests); >100 alcohol use episodes in the past year; major medical problems (e.g., neurological disorder, traumatic brain injury with loss of consciousness >2 min, complicated or premature birth); history of developmental disability; prenatal substance exposure; use of psychoactive medications at project intake; uncorrectable sensory impairments; left-handedness; MRI contraindications (e.g., implanted metal and metal braces); and limited proficiency in English.

Upon confirming eligibility, participants were extended an invitation to visit the laboratory for a 4 h session. This session encompassed various activities, including brain imaging acquisition, the collection of demographic information, neuropsychological assessments, and a substance use assessment with toxicological analysis. Participants were specifically instructed to abstain from cannabis use within the 12 h leading up to their appointment to prevent the acute effects of intoxication. However, they were not required to abstain from NTP use to avoid potential withdrawal effects during data collection.

For this study, and to maintain alignment with prior studies conducted in our laboratory [36], participants were divided into two groups for data analysis (NTP and non-NTP) based on their patterns of past-year NTP use. The NTP use group consisted of individuals who reported an average of more than one episode of NTP use per month in the previous year, with a minimum of 12 episodes of NTP use within the past year. Participants were also divided into two groups (cannabis and non-cannabis) based on their past-year cannabis use patterns. The cannabis use group included those individuals reporting at least one episode of cannabis use per month during the previous year, totaling 12 or more episodes of cannabis use within the past year.

### 2.2. Measures

#### 2.2.1. Demographics and Substance Use

A demographic and psychosocial interview was conducted to assess background information on race/ethnicity, socioeconomic status, education, and medical history. The participants’ history of substance use was gathered utilizing an adapted version of The Customary Drinking and Drug Use Record (CDDR), which was modified to encompass additional inquiries about cannabis and nicotine usage [36,37,43,44]. Specifically, participants were interviewed about instances of independent NTP and cannabis use within the past year. Given the diversity of emerging NTPs, our definition of NTP encompassed a range of products, including traditional tobacco cigarettes, tobacco in pipes, various types of cigars, electronic cigarettes, vape pens, Juul devices, e-hookahs, smokeless tobacco products like chewing tobacco, snuff, or snus, tobacco-based hookah, as well as nicotine replacements such as patches, gums, nasal sprays, inhalers, and lozenges. Additional data regarding the age at which participants initially used these substances and the most recent instance of use were also gathered.

#### 2.2.2. Image Acquisition and Processing

MRI acquisitions were carried out on a 3-Tesla GE DiscoveryMR750 scanner using a Nova medical 32-channel head coil at the University of California, San Diego, Center of Functional MRI. All subjects wore soft earplugs, and foam pads were positioned around the participants’ heads to minimize head movement and reduce scanner noise. T1-weighted anatomical sagittal 3D images were acquired using the following parameters: TI/TE/TR = 1060/2.92/6.952 ms, flip angle = 8°, FOV = 256 mm, acquisition matrix = 256 mm × 256 mm, and slice thickness = 1.0 mm.

T1-weighted structural MRI were analyzed using FreeSurfer 6.0 (http://surfer.nmr.mgh.harvard.edu/; accessed on 1 January 2023). Briefly, the processing included removal of non-brain, Talairach transformation, segmentation of the subcortical white matter and deep gray matter volumetric structures, intensity normalization, tessellation of the gray-matter–white-matter boundary, topology correction, and surface deformation to form the gray/white and gray/CSF boundary [45,46,47]. These surfaces were visually inspected and manually edited by one of the authors, Q.S., who was blind to the subject’s group status. Once the cortical models were completed, several deformable procedures were performed, including surface inflation, registration to a spherical atlas, cortex parcellation, and creation of a variety of surface-based data. Among these, cortical thickness was calculated as the closest distance from the gray/white boundary to the gray/CSF boundary at each vertex on the tessellated surface [45,46,47]. In the current project, we examined cortical thickness according to the Desikan–Killiany atlas provided by FreeSurfer [48] within frontal cortical regions.

### 2.3. Data Analyses

Following imaging post-processing, data analysis was conducted utilizing Rstudio, R version 2023.03.1+446. Independent *t* or Chi-square tests were used to compare group differences in demographic attributes, substance use patterns, and mental health variables. A series of linear regression models were carried out to investigate the independent and interactive effects of nicotine and cannabis group status on 11 a priori bilateral cortical thickness estimates within the frontal lobe. All linear regressions were run using gamm4 package. To account for multiple comparisons, a False Discovery Rate of 0.05 was applied to each test within hemisphere and variable (nicotine, cannabis, and the interaction between nicotine and cannabis) using the p.adjust function in stat package.

## 3. Results

### 3.1. Demographics

Characteristics of NTP users and non-NTP users. Table 1 presents demographic characteristics for NTP users (n = 136) and non-NTP users (n = 87). Within our sample, a substantial majority of participants (61%) reported engaging in one or more NTP use episodes per month over the past year. Among those reporting 12 or more past-year NTP episodes, the average frequency was 2327.2 episodes (SD = 4534.5) in the past year. NTP users were found to be slightly older, with a mean age of 19.7 (SD = 1.5), in contrast to the non-NTP use group, which had a mean age of 19.2 (SD = 1.7), t (166) = −2.24, *p* = 0.03. While the percentage of males showed a trend (41.2% for NTP users vs. 54% for non-NTP users, *p* = 0.08), no significant differences were observed between the two groups in terms of sex, race (White), Hispanic ethnicity, or number of educational years completed. Notably, the percentage of those reporting cannabis use (*p* < 0.01) and the total number of past-year alcohol drinks (*p* < 0.01) were significantly higher among NTP users compared to non-NTP users.

Characteristics of cannabis users and non-cannabis users. In Table 2, we examined and compared the demographic and substance use characteristics of cannabis users (n = 156) and non-cannabis users (n = 67). Cannabis users were, on average, slightly older (19.6 years, SD = 1.5) than non-cannabis users (19.1 years, SD = 1.7), t (166) = −2.04, *p* = 0.04. While a significantly higher percentage of males was observed among non-cannabis users (60%) compared to cannabis users (40%, *p* = 0.01), no statistically significant differences were observed in terms of race (White), Hispanic ethnicity, and educational attainment. Notably, a substantial percentage of cannabis users reported NTP use (76.3%) compared to non-cannabis users (25.4%, *p* < 0.01). Cannabis users also reported a significantly higher total number of past-year alcohol drinks compared to non-cannabis users (*p* < 0.01).

We incorporated age and alcohol use status (past year total drinks) as covariates in our analyses, recognizing their potential influence on our research findings and in line with our demographic observations between groups. However, the analyses revealed non-significant effects of alcohol use on cortical thickness (*p*s > 0.05). Intracranial volume (ICV) was also included as a covariate to control for head size [15,42].

### 3.2. Cortical Thickness Estimates

#### 3.2.1. Right Hemisphere Cortical Thickness Estimates

A significant main effect of nicotine on cortical thickness was detected in the precentral gyrus (β = −0.09, *t* (216) = −2.99, *p* = 0.03), with nicotine users showing thinner cortices than those who did not use nicotine. No other significant main effects of nicotine or cannabis were identified concerning the other regions of interest (see Table 3). The interaction between nicotine and cannabis was not significantly associated with cortical thickness in the right hemisphere (see Table 4).

#### 3.2.2. Left Hemisphere Cortical Thickness Estimates

**Figure 1:**
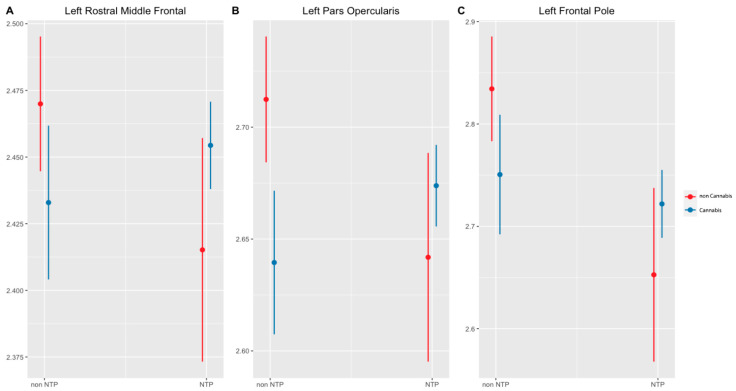
Significant interactions of cortical thickness by Brain region (*p* < 0.05, corrected). Notes: NTP = Nicotine Tobacco Products. Cannabis and NTP group interaction on left rostral middle frontal (**A**) left pars opercularis (**B**) and left frontal pole (**C**) cortical thickness. Cannabis use status had a significant effect on cortical thickness, with cannabis use in the non-NTP group having lower cortical thickness compared to non-cannabis use. Conversely, in the NTP group, cannabis use exhibited higher cortical thickness compared to non-cannabis use.

In the left hemisphere, main effects were observed for nicotine and cannabis on cortical thickness estimates. Nicotine users had thinner gray matter cortices than non-nicotine users in the superior frontal gyrus (β = −0.07, t (216) = −2.53, *p* = 0.04), pars opercularis (β = −0.07, t (216) = −2.54, *p* = 0.04), and frontal pole (β = −0.18, t (216) = −3.59, *p* = 0.00. Concurrently, cannabis use was significantly associated with thinner cortices in the pars opercularis (β = −0.07, t (216) = −3.43, *p* = 0.01) (see Table 3). Significant interactions between nicotine and cannabis were observed in the rostral middle frontal gyrus (*p* = 0.02), pars opercularis (*p* = 0.01), and frontal pole (*p* = 0.04; see Figure 1, Table 4). In these regions, cannabis users without a history of NTP use had thinner cortices than non-cannabis users. Conversely, among those with a history of NTP use, cannabis use was associated with thicker cortices. Notably, individuals abstaining from NTPs demonstrated thinner cortices when using cannabis, contrasting with those not using cannabis. The NTP group exhibited thinner cortices in the absence of cannabis use but exhibited thicker cortices when cannabis use was present.

## 4. Discussion

In summary, our study investigated the neurobiological impact of NTPs and cannabis on cortical thickness in the frontal gray matter of individuals aged 16 to 22 with and without a history of regular (at least monthly) cannabis and nicotine use. We observed that NTP use is linked to thinner cortical estimates in specific regions, including the right precentral gyrus and left superior frontal gyrus. These regional effects suggest a targeted influence of NTP on cortical structures that underlie motor function and executive functions (e.g., right precentral gyrus, left superior frontal gyrus), which aligns with previous research demonstrating increased activation and connectivity in these regions [49,50]. Furthermore, a significant interaction between NTP and cannabis on cortical thickness estimates emerged in the rostral middle frontal gyrus, pars opercularis, and frontal pole. Within these regions, cannabis users exhibited thinner cortices if they did not have a history of NTP use. Conversely, among cannabis users with a history of NTP use, cannabis was linked to thicker cortices. These interactions suggest that the effects of cannabis use on cortical thickness in the left hemisphere may be modulated by a history of NTP use, such that nicotine may counteract possible deleterious effects related to cannabis use or influence the brain with different mechanisms (e.g., atrophy and inflammation). This finding indicates that the relationship between cannabis and brain structure may be influenced by previous exposure to nicotine, similar to other work in our laboratory looking at cannabis and nicotine interactions on metrics of brain integrity [36,37].

Cannabis, primarily through the psychoactive compound delta-9-tetrahydrocannabinol (THC), interacts with the developing endocannabinoid system (ECS) during adolescence, a period marked by ECS maturation and a higher density of CB1 receptors in the brain. Preclinical evidence suggests that the activation of CB1 receptors may play a crucial role in regulating structural brain development, influencing synaptic plasticity and impacting processes like synaptic pruning, dendritic arborization, and synaptogenesis, thereby shaping the brain’s structural organization [51,52]. Studies involving adolescents and emerging adults have reported varying findings regarding the impact of cannabis use [53]. We observed a significant cannabis effect in the left pars opercularis region (i.e., diminished cortical thickness estimates), which aligns with earlier research by Epstein and Kumra [47]. Broadly, observations suggest that there are minimal differences in cortical thickness estimates among cannabis users across various frontal regions of the brain, but cannabis user status is associated with thinner estimates. Yet, among cannabis users with a history of NTP use, we observe a contrasting pattern: thicker estimates, in contrast to thinner estimates for cannabis users who do not report using NTP. Furthermore, while some studies have reported thicker [15,54] and thinner [54,55] cortical thickness estimates among young adult cannabis users in frontal ROIs, many studies did not account for nicotine use. These differences highlight the complex and variable relationship between cannabis use and cortical thickness, particularly for regions rich in CB1 receptors. Consequently, this variability in findings may be influenced by factors related to study design, population characteristics, and methodological considerations.

Nicotine, a key component found in tobacco and electronic cigarettes, intricately engages with the developing brain primarily through its interaction with nicotinic acetylcholine receptors (nAChRs) crucial for regulating cognitive and neural functions, potentially resulting in changes at both structural and functional levels [56,57,58]. Prior research has shown that nicotine exposure during this adolescent and emerging adult developmental phase can influence neural plasticity and neurotransmitter systems, potentially leading to modifications in gray matter integrity, white matter development, and cortical thickness [27,29,59,60,61]. Our findings, showing thinner cortical estimates in regions such as the right precentral gyrus, left superior frontal gyrus, left pars opercularis, and left frontal pole among nicotine users, may reflect deleterious alterations in neuromaturation. Specifically, our results align with previous studies reporting cortical thinning in adolescents and young adults using nicotine, particularly in the left hemisphere regions like the superior frontal gyrus and pars opercularis; however, we did not observe significant differences in other regions [27].

While prior studies have explored how cannabis and NTPs can independently influence brain structure, the neurobiological impact of their combined use is even more intricate and multifaceted. This complexity arises from the interactions of both substances on neurotransmitters and receptors, which may lead to alterations in macrostructural development. For example, nicotine may stimulate the ECS, releasing anandamide and 2-AG and modulating the brain’s reward circuity, and thereby influencing dopamine levels and enhancing the rewarding effects of nicotine [26,62]. It appears that the relationship between cannabis and brain structure may be modulated by prior exposure to nicotine, as nicotine may potentially counteract some of the deleterious effects associated with cannabis use. This interaction suggests that the interplay between NTPs and cannabis extends beyond mere additive effects and involves intricate modulations within the brain’s neural networks. Prior research from our laboratory indicates the presence of a distinctive white matter structure among co-users, as compared to cannabis-only users, [37], which may be attributed to nicotine’s activation of nAChRs and its role in enhancing glial activity. Nicotine is recognized for its ability to stimulate the release of neurotransmitters such as dopamine and norepinephrine [63]. Additionally, it can elevate acetylcholine levels, which may enhance cognitive function [56,64,65]. These effects could potentially offset some of the adverse cognitive impacts of cannabis. The interplay between nicotine and cannabis may also exhibit individual variability, and such variability in findings may be attributed to factors such as the duration and frequency of cannabis use, age of onset, participant population studies, genetic and environmental factors, individual differences, and specific type of tobacco/cannabis product used [3,11,66].

While this study presents valuable insights into the relationship of NTP and cannabis co-use with cortical development among young adults, several limitations should be acknowledged. Importantly, the study’s cross-sectional design impedes the establishment of causal relationships, and the reliance on self-reported substance use could introduce recall bias or underreporting, potentially affecting the accuracy of substance use patterns reported. The focus solely on co-use within the past year may overlook lifelong cumulative effects, as compared to recency of use, and the study’s concentration on frontal cortical regions and cortical thickness does not provide comprehensive data on gray matter changes that may be related to co-use in other subcortical brain regions and brain metrics of health. Our study did not include cognitive tasks performed by cannabis and NTP users; future studies should consider incorporating cognitive tasks to further elucidate this relationship. While alcohol was not found to be a significant predictor of cortical thickness in our study, future research should expand this work to better understand the degree to which alcohol use influences nicotine and cannabis co-use-related brain imaging outcomes. Furthermore, a nuanced exploration of nicotine dose combined with objective markers of nicotine use via biological specimen assessment (e.g., hair and blood toxicology) could enhance our understanding of the intricate interplay between nicotine and cannabis across diverse brain regions and health metrics.

## 5. Conclusions

In summary, our study finds unique effects of NTPs and cannabis on frontal brain cortical thickness estimates. Furthermore, it highlights a nuanced and intricate interaction between cannabis and nicotine in the developing brain, and unique gray matter phenotypes related to single substance use as compared to co-use. Future studies that use comprehensive longitudinal designs will continue to probe the effects of cannabis and nicotine use on brain development.

## Figures and Tables

**Table 1:** Demographic and substance use characteristics of Nicotine and Tobacco Product users (NTP) and NTP non-users (non-NTP).

	Group [Mean (Standard Deviation) or %]		
Variable	Non-NTP	NTP	*p*-Value
Total N	n = 87	n = 136	
Age	19.2 (1.7)	19.7 (1.5)	**0.03**
% Male	54%	41.2%	0.08
% White	48.3%	51.5%	0.74
% Hispanic	36.8%	39%	0.85
Education years completed	12.9 (1.6)	13.2 (1.4)	0.15
Nicotine and Tobacco Products			
Days since last used	133.8 (461.9)	35.7 (128.9)	**0.02**
Past year total use episodes	1.7 (3.0)	2327.3 (4534.5)	**<0.01**
% Cannabis Use	42.5%	87.5%	**<0.01**
Past year total alcohol drinks	22.5 (31.1)	61.3 (56.2)	**<0.01**

*p* < 0.05 bold.

**Table 2:** Demographic and substance use characteristics of cannabis users (Cannabis) and non-users (Non-Cannabis).

	Group [Mean (Standard Deviation) or %]		
Variable	Non-Cannabis	Cannabis	*p*-Value
Total N	n = 67	n = 156	
Age	19.1 (1.7)	19.6 (1.5)	**0.04**
% Male	60%	40%	**0.01**
% Hispanic	31%	41%	0.23
Race	47.8%	51.3%	0.74
Education years completed	12.9 (1.8)	13.2 (1.4)	0.38
Cannabis			
Days since last used	68.8 (176.2)	9.1 (26.4)	0.09
Past year total use episodes	1.7 (3.2)	374.6 (511.4)	**<0.01**
% NTP Use	25.4%	76.3%	**<0.01**
Past year total drinks	25.4 (37.2)	55.1 (54.3)	**<0.01**

*p* < 0.05 bold.

**Table 3:** Main Effects of nicotine and tobacco product (NTP) use and cannabis use. Results represent thinner estimates (mm^3^) in both cannabis and nicotine use groups.

	NTP			Cannabis		
Right Hemisphere	Beta	*p*-Value	Adjusted *p*-Value *	Beta	*p*-Value	Adjusted *p*-Value *
Superior Frontal	−0.06	0.06	0.12	−0.04	0.08	0.27
Rostral Middle Frontal	−0.03	0.18	0.25	−0.03	0.13	0.27
Caudal Middle Frontal	−0.06	0.06	0.12	−0.03	0.26	0.36
Pars Opercularis	−0.07	0.05	0.12	−0.05	**0.03**	0.27
Pars Triangularis	−0.05	0.10	0.16	−0.02	0.44	0.52
Pars Orbitalis	−0.07	0.06	0.12	−0.04	0.15	0.27
Lateral Orbitofrontal	−0.06	**0.04**	0.12	−0.04	0.11	0.27
Medial Orbitofrontal	−0.02	0.47	0.52	−0.03	0.24	0.36
Precentral	−0.09	**0.00**	**0.03**	−0.04	0.06	0.27
Paracentral	−0.03	0.41	0.50	0.00	0.94	0.94
Frontal Pole	0.00	1.00	1.00	−0.03	0.48	0.52
Left Hemisphere						
Superior Frontal	−0.07	**0.01**	**0.04**	−0.06	0.01	0.07
Rostral Middle Frontal	−0.05	**0.03**	0.08	−0.04	0.05	0.12
Caudal Middle Frontal	−0.04	0.19	0.29	−0.04	0.11	0.17
Pars Opercularis	−0.07	**0.01**	**0.04**	−0.07	**0.00**	**0.01**
Pars Triangularis	−0.06	0.06	0.11	−0.03	0.22	0.30
Pars Orbitalis	0.00	0.89	0.98	−0.01	0.63	0.69
Lateral Orbitofrontal	−0.01	0.82	0.98	−0.05	0.03	0.09
Medial Orbitofrontal	−0.02	0.52	0.71	−0.04	0.10	0.17
Precentral	−0.05	0.06	0.11	−0.02	0.37	0.45
Paracentral	0.00	0.99	0.99	0.00	1.00	1.00
Frontal Pole	−0.18	**0.00**	**0.00**	−0.08	**0.03**	0.09

* Adjusted for Multiple Comparisons within Hemisphere and Substance: False Discovery Rate. *p* < 0.05 bold.

**Table 4:** Interactive effects of nicotine and tobacco product (NTP) use and cannabis use. Among those with a history of NTP use, cannabis use was associated with thicker cortices (mm^3^).

	Right Hemisphere			Left Hemisphere		
Brain Region	Beta	*p*-Value	Adjusted *p*-Value *	Beta	*p*-Value	Adjusted *p*-Value *
Superior Frontal	0.07	0.07	0.17	0.07	**0.04**	0.10
Rostral Middle Frontal	0.05	0.10	0.18	0.08	**0.01**	**0.04**
Caudal Middle Frontal	0.06	0.08	0.17	0.05	0.15	0.26
Pars Opercularis	0.09	**0.03**	0.09	0.10	**0.00**	**0.02**
Pars Triangularis	0.03	0.37	0.49	0.06	0.09	0.19
Pars Orbitalis	0.10	**0.02**	0.09	0.03	0.52	0.57
Lateral Orbitofrontal	0.05	0.12	0.18	0.04	0.29	0.39
Medial Orbitofrontal	0.02	0.53	0.58	0.03	0.36	0.44
Precentral	0.10	**0.00**	0.05	0.04	0.16	0.26
Paracentral	0.03	0.40	0.49	0.00	0.92	0.92
Frontal Pole	0.02	0.78	0.78	0.15	**0.01**	**0.04**

* Adjusted for Multiple Comparisons within Hemisphere: False Discovery Rate = 0.05. *p* < 0.05 bold.

## Data Availability

Data are not publicly available due to restrictions (e.g., their containing information that could compromise the privacy of research participants) but are available upon request from the authors.

## References

[1] Miech R.A., Johnston L.D., O’Malle P.M., Patrick M.E., Bachman J.G., Schulenberg J.E. (2023). Monitoring the Future National Survey Results on Drug Use, 1975–2022: Secondary School Students.

[2] Patrick M.E., Miech R.A., Johnston L.D., O’Malley P.M. (2023). Monitoring the Future Panel Study Annual Report: National Data on Substance Use among Adults Ages 19 to 60, 1976–2022.

[3] Agrawal A., Budney A.J., Lynskey M.T. (2012). The Co-Occurring Use and Misuse of Cannabis and Tobacco: A Review. Addiction.

[4] Chu A., Chaiton M., Kaufman P., Goodwin R.D., Lin J., Hindocha C., Goodman S., Hammond D. (2023). Co-Use, Simultaneous Use, and Mixing of Cannabis and Tobacco: A Cross-National Comparison of Canada and the US by Cannabis Administration Type. Int. J. Environ. Res. Public Health.

[5] Goodwin R.D., Pacek L.R., Copeland J., Moeller S.J., Dierker L., Weinberger A., Gbedemah M., Zvolensky M.J., Wall M.M., Hasin D.S. (2018). Trends in Daily Cannabis Use Among Cigarette Smokers: United States, 2002–2014. Am. J. Public Health.

[6] Hindocha C., Freeman T.P., Ferris J.A., Lynskey M.T., Winstock A.R. (2016). No Smoke without Tobacco: A Global Overview of Cannabis and Tobacco Routes of Administration and Their Association with Intention to Quit. Front. Psychiatry.

[7] Pacek L.R., Copeland J., Dierker L., Cunningham C.O., Martins S.S., Goodwin R.D. (2018). Among Whom Is Cigarette Smoking Declining in the United States? The Impact of Cannabis Use Status, 2002–2015. Drug Alcohol Depend..

[8] Schaefer J.D., Jang S.-K., Clark D.A., Deak J.D., Hicks B.M., Iacono W.G., Liu M., McGue M., Vrieze S.I., Wilson S. (2023). Associations between Polygenic Risk of Substance Use and Use Disorder and Alcohol, Cannabis, and Nicotine Use in Adolescence and Young Adulthood in a Longitudinal Twin Study. Psychol. Med..

[9] Smith D.M., Miller C., O’Connor R.J., Kozlowski L.T., Wadsworth E., Fix B.V., Collins R.L., Wei B., Goniewicz M.L., Hyland A.J. (2021). Modes of Delivery in Concurrent Nicotine and Cannabis Use (“Co-Use”) among Youth: Findings from the International Tobacco Control (Itc) Survey. Subst. Abuse.

[10] Weinberger A.H., Pacek L.R., Wall M.M., Zvolensky M.J., Copeland J., Galea S., Nahvi S., Moeller S.J., Hasin D.S., Goodwin R.D. (2018). Trends in Cannabis Use Disorder by Cigarette Smoking Status in the United States, 2002–2016. Drug Alcohol Depend..

[11] Cobb C.O., Soule E.K., Rudy A.K., Sutter M.E., Cohn A.M. (2018). Patterns and Correlates of Tobacco and Cannabis Co-Use by Tobacco Product Type: Findings from the Virginia Youth Survey. Subst. Use Misuse.

[12] Nguyen N., Barrington-Trimis J.L., Urman R., Cho J., McConnell R., Leventhal A.M., Halpern-Felsher B. (2019). Past 30-Day Co-Use of Tobacco and Marijuana Products among Adolescents and Young Adults in California. Addict. Behav..

[13] Bava S., Thayer R., Jacobus J., Ward M., Jernigan T.L., Tapert S.F. (2010). Longitudinal Characterization of White Matter Maturation during Adolescence. Brain Res..

[14] Brown T.T., Kuperman J.M., Chung Y., Erhart M., McCabe C., Hagler D.J., Venkatraman V.K., Akshoomoff N., Amaral D.G., Bloss C.S. (2012). Neuroanatomical Assessment of Biological Maturity. Curr. Biol. CB.

[15] Jacobus J., Squeglia L.M., Meruelo A.D., Castro N., Brumback T., Giedd J.N., Tapert S.F. (2015). Cortical Thickness in Adolescent Marijuana and Alcohol Users: A Three-Year Prospective Study from Adolescence to Young Adulthood. Dev. Cogn. Neurosci..

[16] Jernigan T.L., Baaré W.F.C., Stiles J., Madsen K.S. (2011). Postnatal Brain Development: Structural Imaging of Dynamic Neurodevelopmental Processes. Prog. Brain Res..

[17] Palmer C.E., Pecheva D., Iversen J.R., Hagler D.J., Sugrue L., Nedelec P., Fan C.C., Thompson W.K., Jernigan T.L., Dale A.M. (2021). Microstructural Development from 9 to 14 Years: Evidence from the ABCD Study. Dev. Cogn. Neurosci..

[18] Sowell E.R., Trauner D.A., Gamst A., Jernigan T.L. (2002). Development of Cortical and Subcortical Brain Structures in Childhood and Adolescence: A Structural MRI Study. Dev. Med. Child Neurol..

[19] Tamnes C.K., Herting M.M., Goddings A.-L., Meuwese R., Blakemore S.-J., Dahl R.E., Güroğlu B., Raznahan A., Sowell E.R., Crone E.A. (2017). Development of the Cerebral Cortex across Adolescence: A Multisample Study of Inter-Related Longitudinal Changes in Cortical Volume, Surface Area, and Thickness. J. Neurosci..

[20] Wierenga L.M., Langen M., Oranje B., Durston S. (2014). Unique Developmental Trajectories of Cortical Thickness and Surface Area. NeuroImage.

[21] Galvan A., Hare T.A., Parra C.E., Penn J., Voss H., Glover G., Casey B.J. (2006). Earlier Development of the Accumbens Relative to Orbitofrontal Cortex Might Underlie Risk-Taking Behavior in Adolescents. J. Neurosci..

[22] Sharma A., Morrow J.D. (2016). Neurobiology of Adolescent Substance Use Disorders. Child Adolesc. Psychiatr. Clin. N. Am..

[23] Jacobus J., McQueeny T., Bava S., Schweinsburg B.C., Frank L.R., Yang T.T., Tapert S.F. (2009). White Matter Integrity in Adolescents with Histories of Marijuana Use and Binge Drinking. Neurotoxicol. Teratol..

[24] Jacobus J., Squeglia L.M., Bava S., Tapert S.F. (2013). White Matter Characterization of Adolescent Binge Drinking with and without Co-Occurring Marijuana Use: A 3-Year Investigation. Psychiatry Res..

[25] Jacobus J., Squeglia L.M., Infante M.A., Bava S., Tapert S.F. (2013). White Matter Integrity Pre- and Post Marijuana and Alcohol Initiation in Adolescence. Brain Sci..

[26] Subramaniam P., McGlade E., Yurgelun-Todd D. (2016). Comorbid Cannabis and Tobacco Use in Adolescents and Adults. Curr. Addict. Rep..

[27] Akkermans S.E.A., van Rooij D., Rommelse N., Hartman C.A., Hoekstra P.J., Franke B., Mennes M., Buitelaar J.K. (2017). Effect of Tobacco Smoking on Frontal Cortical Thickness Development: A Longitudinal Study in a Mixed Cohort of ADHD-Affected and -Unaffected Youth. Eur. Neuropsychopharmacol..

[28] Chye Y., Mackey S., Gutman B.A., Ching C.R.K., Batalla A., Blaine S., Brooks S., Caparelli E., Cousijn J., Dagher A. (2020). Subcortical Surface Morphometry in Substance Dependence: An ENIGMA Addiction Working Group Study. Addict. Biol..

[29] Li Y., Yuan K., Cai C., Feng D., Yin J., Bi Y., Shi S., Yu D., Jin C., von Deneen K.M. (2015). Reduced Frontal Cortical Thickness and Increased Caudate Volume within Fronto-Striatal Circuits in Young Adult Smokers. Drug Alcohol Depend..

[30] Xiang S., Jia T., Xie C., Cheng W., Chaarani B., Banaschewski T., Barker G.J., Bokde A.L.W., Büchel C., Desrivières S. (2023). Association between vmPFC Gray Matter Volume and Smoking Initiation in Adolescents. Nat. Commun..

[31] Filbey F.M., McQueeny T., Kadamangudi S., Bice C., Ketcherside A. (2015). Combined Effects of Marijuana and Nicotine on Memory Performance and Hippocampal Volume. Behav. Brain Res..

[32] Rabin R.A., George T.P. (2015). A Review of Co-Morbid Tobacco and Cannabis Use Disorders: Possible Mechanisms to Explain High Rates of Co-Use. Am. J. Addict..

[33] Jacobsen L.K., Pugh K.R., Constable R.T., Westerveld M., Mencl W.E. (2007). Functional Correlates of Verbal Memory Deficits Emerging during Nicotine Withdrawal in Abstinent Adolescent Cannabis Users. Biol. Psychiatry.

[34] Schuster R.M., Crane N.A., Mermelstein R., Gonzalez R. (2015). Tobacco May Mask Poorer Episodic Memory among Young Adult Cannabis Users. Neuropsychology.

[35] Schuster R.M., Mermelstein R.J., Hedeker D. (2016). Ecological Momentary Assessment of Working Memory Under Conditions of Simultaneous Marijuana and Tobacco Use. Addiction.

[36] Courtney K.E., Baca R., Doran N., Jacobson A., Liu T.T., Jacobus J. (2020). The Effects of Nicotine and Cannabis Co-Use during Adolescence and Young Adulthood on White Matter Cerebral Blood Flow Estimates. Psychopharmacology.

[37] Courtney K.E., Sorg S., Baca R., Doran N., Jacobson A., Liu T.T., Jacobus J. (2022). The Effects of Nicotine and Cannabis Co-Use During Late Adolescence on White Matter Fiber Tract Microstructure. J. Stud. Alcohol Drugs.

[38] Wetherill R.R., Jagannathan K., Hager N., Childress A.R., Rao H., Franklin T.R. (2015). Cannabis, Cigarettes, and Their Co-Occurring Use: Disentangling Differences in Gray Matter Volume. Int. J. Neuropsychopharmacol..

[39] Mejia M.H., Wade N.E., Baca R., Diaz V.G., Jacobus J. (2021). The Influence of Cannabis and Nicotine Co-Use on Neuromaturation—A Systematic Review of Adolescent and Young Adult Studies. Biol. Psychiatry.

[40] Giedd J.N. (2008). The Teen Brain: Insights from Neuroimaging. J. Adolesc. Health.

[41] Infante M.A., Courtney K.E., Castro N., Squeglia L.M., Jacobus J. (2018). Adolescent Brain Surface Area Pre- and Post-Cannabis and Alcohol Initiation. J. Stud. Alcohol Drugs.

[42] Jacobus J., Castro N., Squeglia L.M., Meloy M.J., Brumback T., Huestis M., Tapert S.F. (2016). Adolescent Cortical Thickness Pre- and Post Marijuana and Alcohol Initiation. Neurotoxicol. Teratol..

[43] Brown S.A., Myers M.G., Lippke L., Tapert S.F., Stewart D.G., Vik P.W. (1998). Psychometric Evaluation of the Customary Drinking and Drug Use Record (CDDR): A Measure of Adolescent Alcohol and Drug Involvement. J. Stud. Alcohol.

[44] Wade N.E., Baca R., Courtney K.E., McCabe C.J., Infante M.A., Huestis M.A., Jacobus J. (2021). Preliminary Evidence for Cannabis and Nicotine Urinary Metabolites as Predictors of Verbal Memory Performance and Learning Among Young Adults. J. Int. Neuropsychol. Soc. JINS.

[45] Dale A.M., Fischl B., Sereno M.I. (1999). Cortical Surface-Based Analysis. I. Segmentation and Surface Reconstruction. NeuroImage.

[46] Fischl B., Sereno M.I., Dale A.M. (1999). Cortical Surface-Based Analysis. II: Inflation, Flattening, and a Surface-Based Coordinate System. NeuroImage.

[47] Fischl B., Dale A.M. (2000). Measuring the Thickness of the Human Cerebral Cortex from Magnetic Resonance Images. Proc. Natl. Acad. Sci. USA.

[48] Desikan R.S., Ségonne F., Fischl B., Quinn B.T., Dickerson B.C., Blacker D., Buckner R.L., Dale A.M., Maguire R.P., Hyman B.T. (2006). An Automated Labeling System for Subdividing the Human Cerebral Cortex on MRI Scans into Gyral Based Regions of Interest. NeuroImage.

[49] Addicott M.A., Sweitzer M.M., McClernon F.J. (2018). The Effects of Nicotine and Tobacco Use on Brain Reward Function: Interaction with Nicotine Dependence Severity. Nicotine Tob. Res..

[50] Tan Y., Chen J., Liao W., Qian Z. (2019). Brain Function Network and Young Adult Smokers: A Graph Theory Analysis Study. Front. Psychiatry.

[51] González S., Cebeira M., Fernández-Ruiz J. (2005). Cannabinoid Tolerance and Dependence: A Review of Studies in Laboratory Animals. Pharmacol. Biochem. Behav..

[52] Orihuel J., Capellán R., Casquero-Veiga M., Soto-Montenegro M.L., Desco M., Oteo-Vives M., Ibáñez-Moragues M., Magro-Calvo N., Luján V.M., Morcillo M.Á. (2023). The Long-Term Effects of Adolescent Δ9-Tetrahydrocannabinol on Brain Structure and Function Assessed through Neuroimaging Techniques in Male and Female Rats. Eur. Neuropsychopharmacol..

[53] Lichenstein S.D., Manco N., Cope L.M., Egbo L., Garrison K.A., Hardee J., Hillmer A.T., Reeder K., Stern E.F., Worhunsky P. (2022). Systematic Review of Structural and Functional Neuroimaging Studies of Cannabis Use in Adolescence and Emerging Adulthood: Evidence from 90 Studies and 9441 Participants. Neuropsychopharmacology.

[54] Lopez-Larson M.P., Bogorodzki P., Rogowska J., McGlade E., King J.B., Terry J., Yurgelun-Todd D. (2011). Altered Prefrontal and Insular Cortical Thickness in Adolescent Marijuana Users. Behav. Brain Res..

[55] Lisdahl K.M., Tamm L., Epstein J.N., Jernigan T., Molina B.S.G., Hinshaw S.P., Swanson J.M., Newman E., Kelly C., Bjork J.M. (2016). The Impact of ADHD Persistence, Recent Cannabis Use, and Age of Regular Cannabis Use Onset on Subcortical Volume and Cortical Thickness in Young Adults. Drug Alcohol Depend..

[56] Valentine G., Sofuoglu M. (2018). Cognitive Effects of Nicotine: Recent Progress. Curr. Neuropharmacol..

[57] Yuan M., Cross S.J., Loughlin S.E., Leslie F.M. (2015). Nicotine and the Adolescent Brain. J. Physiol..

[58] Ren M., Lotfipour S., Leslie F. (2022). Unique Effects of Nicotine across the Lifespan. Pharmacol. Biochem. Behav..

[59] Chaarani B., Kan K.-J., Mackey S., Spechler P.A., Potter A., Orr C., D’Alberto N., Hudson K.E., Banaschewski T., Bokde A.L.W. (2019). Low Smoking Exposure, the Adolescent Brain, and the Modulating Role of CHRNA5 Polymorphisms. Biol. Psychiatry Cogn. Neurosci. Neuroimaging.

[60] Conti A.A., Baldacchino A.M. (2022). Chronic Tobacco Smoking, Impaired Reward-Based Decision-Making, and Role of Insular Cortex: A Comparison between Early-Onset Smokers and Late-Onset Smokers. Front. Psychiatry.

[61] Morales A.M., Ghahremani D., Kohno M., Hellemann G.S., London E.D. (2014). Cigarette Exposure, Dependence, and Craving Are Related to Insula Thickness in Young Adult Smokers. Neuropsychopharmacology.

[62] Scherma M., Muntoni A.L., Melis M., Fattore L., Fadda P., Fratta W., Pistis M. (2016). Interactions between the Endocannabinoid and Nicotinic Cholinergic Systems: Preclinical Evidence and Therapeutic Perspectives. Psychopharmacology.

[63] Benowitz N.L. (2009). Pharmacology of Nicotine: Addiction, Smoking-Induced Disease, and Therapeutics. Annu. Rev. Pharmacol. Toxicol..

[64] Tiwari R.K., Sharma V., Pandey R.K., Shukla S.S. (2020). Nicotine Addiction: Neurobiology and Mechanism. J. Pharmacopunct..

[65] Campos M.W., Serebrisky D., Castaldelli-Maia J.M. (2016). Smoking and Cognition. Curr. Drug Abuse Rev..

[66] Ramo D.E., Liu H., Prochaska J.J. (2012). Tobacco and Marijuana Use among Adolescents and Young Adults: A Systematic Review of Their Co-Use. Clin. Psychol. Rev..

